# Traversing the drug discovery landscape using native mass spectrometry

**DOI:** 10.1016/j.sbi.2025.102993

**Published:** 2025-02-01

**Authors:** Hannah M. Britt, Carol V. Robinson

**Affiliations:** 1 Department of Chemistry, University of Oxford, South Parks Road, Oxford, OX1 3TA, UK; 2 Kavli Institute for Nanoscience Discovery, Dorothy Crowfoot Hodgkin Building, Oxford, OX1 3QU, UK

## Abstract

As health needs in our society evolve, the field of drug discovery must undergo constant innovation and improvement to identify novel targets and drug candidates. Owing to its ability to simultaneously capture biological interactions and provide in-depth molecular characterisation of the species involved, native mass spectrometry is starting to play an important role in this endeavour. Here, we discuss recent contributions that native mass spectrometry has made to drug discovery including deciphering protein-small molecule interactions, unravelling biochemical pathways, and integrating with complementary structural approaches.

## Introduction

Since its inception, the field of drug discovery has sought to identify and validate molecules that have the potential to modulate disease [1]. By successfully employing a development pipeline, the field has brought numerous safe and effective treatments to patients. As the landscape continues to evolve, however, innovative methods are needed to address emerging challenges.

**Figure 1:**
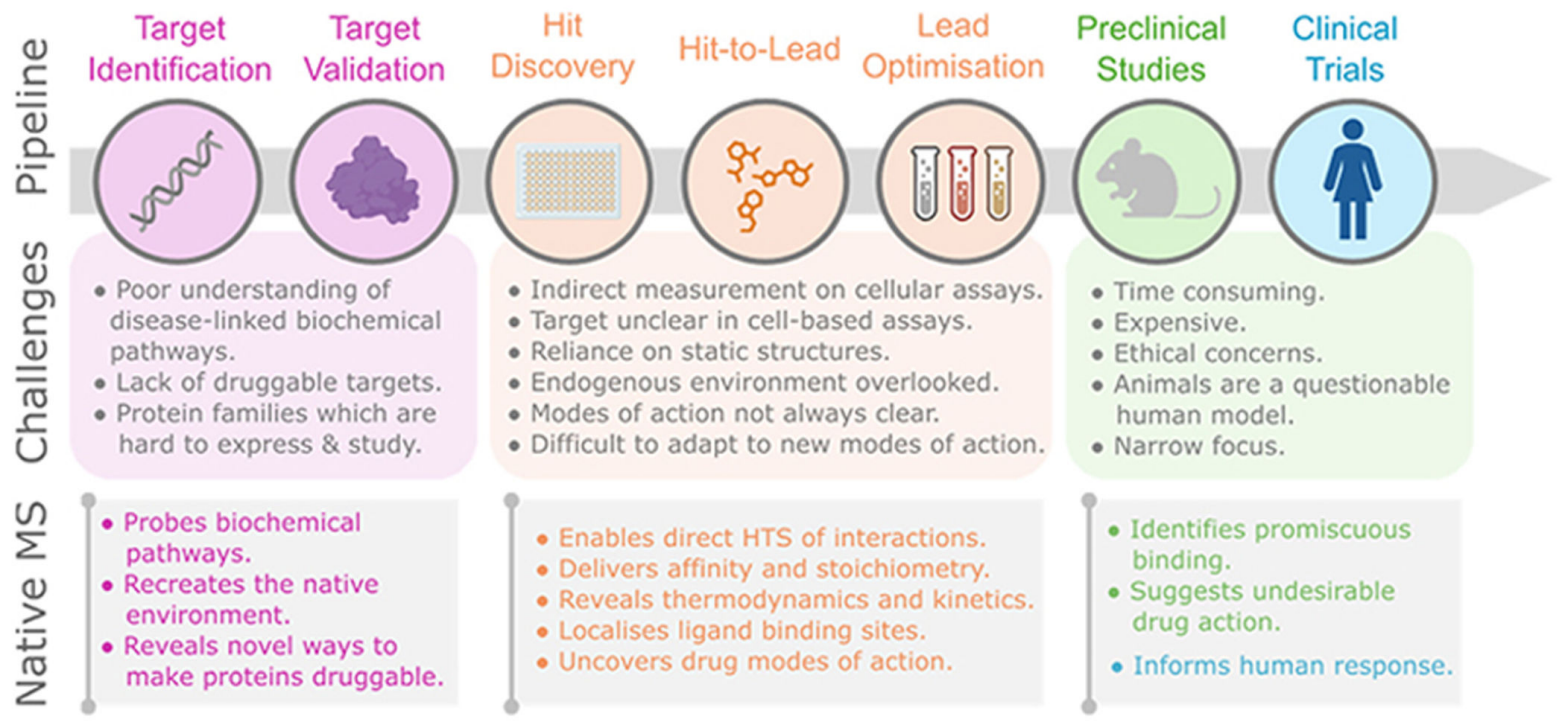
Schematic showing key steps in a typical drug discovery pipeline. Current challenges are associated with each stage and areas where nMS is being applied to address these challenges are highlighted below. Figure created using Biorender.

Native mass spectrometry (nMS) is one approach assisting in overcoming challenges across the drug discovery pipeline. This methodology has been contributing to drug discovery since the early 2000s; specific developments are highlighted in Figure 1. Briefly, in native MS proteins are introduced from solution into the gas phase whilst maintaining many of their endogenous properties [2]. When performed with state-of-the-art instrumentation, at high resolution on a millisecond timescale, only microlitre quantities of nano-to picomolar protein solutions are required. As such, direct and in-depth interrogation of protein non-covalent interactions and native structures is possible, even within heterogeneous populations. Moreover, there is no obvious size limit since globular, glycosylated and disordered protein assemblies, up to 18 MDa in mass, have been characterised [2]. Having overcome early criticism regarding relevance of the gas phase to proteins in solution, nMS now also exploits the solvent-free environment to capture the native properties of membrane proteins [3].

In this review, we discuss recent highlights in nMS-assisted small molecule drug discovery from the last two to three years. Due to space limitations, we are unable to consider the role of nMS in related areas, such as adeno-associated virus (AAV) and lipid delivery systems, vaccine analysis and antibody-based therapies. Instead, we focus on the latest advances and new avenues for exploration that nMS offers across the drug development pipeline. We also comment on currently unexploited areas that we believe will find application for nMS in small molecule drug design.

### Unravelling biochemical pathways

The earliest steps in the drug discovery process involve target identification and validation. These processes focus on unpicking disease mechanisms and identifying protein candidates for drug targeting. They form a crucial step, with some arguing that poor understanding of underlying biology is the principal cause of drug failure [4]. As such, unravelling disease-linked biochemical pathways, and diversifying targets within an environment that accurately reflects their biological context, are critical goals in drug discovery.

The need to unpick enzyme mechanisms in healthy and disease states provide routes for nMS to contribute to the validation of targets. Of relevance here are the insights gleaned from differences between proteoforms, post-translational (PTM) variants and conformational states. These deductions could provide alternative targets that are highly synergistic with current cell-based assays. One area with exciting potential lies in ongoing efforts to replicate the endogenous environment within nMS experiments. We predict that studying proteins within these more native-like environments could assist in addressing the lack of proteins currently considered druggable, i.e. disease-linked and able to be modulated by small molecules. At present, this accounts for only 3%−10% of the proteome. Several protein families are overlooked due to difficulties in studying them outside their native environment, for example orphan receptors, dynamic proteins and membrane complexes.

Latest efforts to replicate native contexts include the pioneering method of protein overexpression followed by nMS analysis of the cell milieu [5]. By applying this workflow to a range of proteins and expression systems, it has been possible to dissect native protein–protein interactions (PPIs) *in situ*, arguably better capturing endogenous protein behaviour. Further expanding the ability to perform nMS in more native-like contexts, nano-desorption electrospray ionisation (DESI) has made possible the ejection of proteins directly from the lens, liver and brain tissues [6–8]. This approach has the additional benefit of offering protein localisation abilities not often associated with nMS. In exciting developments for drug discovery, drug–protein interactions have been maintained in native-like environments using these workflows, enabling molecular targeting of Bruton’s tyrosine kinase and bezafibrate-dosed rats [9,10].

**Figure 2:**
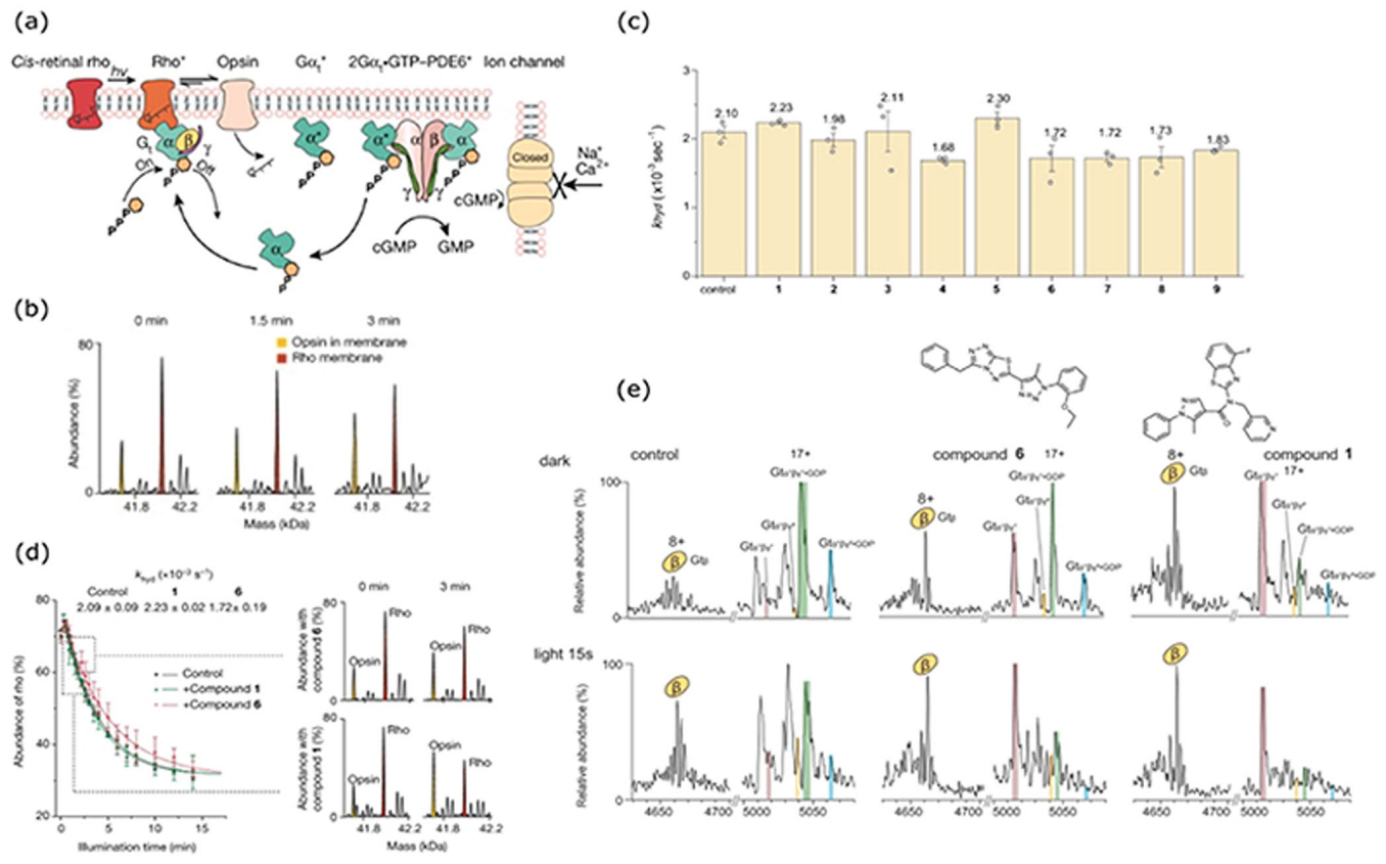
(**a**) Schematic showing key steps in the rhodopsin signalling cascade; **(b)** changes to the rhodopsin to opsin ratio over time in a native membrane following exposure to light; **(c)** rate of hydrolysis comparison (k_hyd_) for rhodopsin in its native membrane in the presence of no compound (control) compared to nine rho-targeting molecules identified in cell-based assays; **(d)** conversion of rhodopsin to opsin after light illumination in the presence of compounds 6 (top) and 1 (below) monitored over time in order to determine binding events and calculate k_hyd_; **(e)** native mass spectra of G-protein signalling activity in the dark (top) and after 15 s of light illumination (bottom) for a control sample compared to in the presence of compounds 6 (middle) and 1 (right). Figure adapted with permission from Chen, S.; Getter, T.; Salom, D.; Wu, D.; Quetschlich, D.; Chorev, D. S.; Palczewski, K.; Robinson, C. V. Capturing a Rhodopsin Receptor Signalling Cascade across a Native Membrane. Nature 2022, 604 (7905), 384–390.

In parallel to these advances, proteoliposomes and sonicated lipid vesicles (SoLVe) have been developed to enable nMS analysis of membrane proteins in their native environment [11–13]. Given that this protein class comprises approximately 60% of clinically approved drug targets, replicating the lipid bilayer holds great potential for informing drug discovery. In an exciting breakthrough in 2022, the signalling cascade of class A G-protein-coupled receptor (GPCR) rhodopsin was captured in real time across its native bovine lens membrane [14]. Within this biological context, photoconversion of cis-retinal bound rhodopsin through its activated rhodopsin* intermediate into its final product opsin could be monitored by its change in mass, Figure 2(b). By monitoring chromophore hydrolysis over time, a rate constant (k_hyd_) for the signalling step could be derived. The team also monitored how the k_hyd_ value was perturbed in the presence of nine known rho-targeting molecules identified from cell-based assays. The results showed that rho ligands could be divided into two distinct functional classes, those that accelerated the rate of conversion, and those that slowed it down Figure 2(c). By focussing in on compounds 1 and 6, from the accelerating and decelerating classes, respectively, it was possible to better understand this modulation, Figure 2(d). Neither compound displayed evidence of changes to rhodopsin conformation or retinal displacement in their nMS spectra, prompting speculation that both molecules operate as allosteric modulators.

Subsequent steps in rhodopsin’s signalling cascade required supplementing the membrane fraction with a soluble preparation containing the downstream signalling components. A rapid reduction in the abundance of the G_t_-GDP complex was observed in the first 15 s following illumination, Figure 2(e); an apo state was formed, which allowed release of the GTP-bound α subunit. That subunit then bound the PDE6 complex, triggering the release of cGMP for ion channel modulation. The exquisite detail in which these steps could be captured, down to the presence of at least two native isoforms of α and γ G-proteins, provided a comprehensive understanding of this biochemical pathway within its endogenous environment. How these steps were modulated by rho-targeting compounds 1 and 6, when compared to a control, showed that both molecules triggered higher levels of G_t_-GDP conversion into its apo form within 15 s of illumination, Figure 2(e). Interestingly, in the presence of either compound, these signalling steps were able to proceed in the dark prior to illumination, attributed to their enhanced isomerisation rates. These combined findings suggest a mode of action where 1 and 6 amplify signalling through G_t_, providing key mechanistic understanding that could be used to inform drug design.

This ability to unravel biochemical pathways across a native membrane in the presence of effectors provides an exciting proof of concept with implications for target identification, validation, and modulation. Key to further advances will be new developments in nMS instrumentation. The ability to not only observe these rhodopsin signalling complexes but also to dissect molecular characteristics of the components is required for less well-characterised signalling pathways. Such endeavours require increasingly sensitive instrumentation capable of multiple rounds of activation, alongside efficient top-down protein fragmentation techniques to better disentangle proteoforms [15,16].

### Modes of action

Principles that have enabled nMS to unravel biochemical pathways are equally applicable to understanding modes of action of pharmaceuticals. This knowledge is the key in connecting drug binding to function, and in so doing predicting how humans might respond to a drug candidate, enabling informed decisions about toxicity and efficacy. nMS has been used to characterise a diverse range of modes of action, including (ant)agonists, drugs that induce protein cleavage or structural changes, and PPI disruptors. In the interests of space, however, we will limit our discussion here to developments in the most novel drug classes.

Proteolysis targeting chimeras (PROTACs) are bifunctional drug candidates that bring a target protein into close contact with an E3 ligase, promoting its polyubiquitination for degradation. Visualising the entire equilibrium of PROTAC complexes in a single MS experiment, has proved insightful in understanding modes of action [17,18]. An excellent example is that of PROTAC MZ1, which induces targeted degradation of protein BRD4 [19]. Interestingly, MZ1 ejection was observed from a compact ternary complex leaving a BRD4-ligase dimer. These observations support a mode of action where the PROTAC facilitates direct contact between the two protein species in order to kickstart the degradation process. Similar approaches are applicable to studying molecular glues, a class of drugs that act to stabilise PPIs [20]. One particular report concluded that aldehyde-based molecular glue (MG1) acts to stabilise the 14-3-3:Pin1 complex in a two-step mechanism regulated by an initial non-covalent event [21–23]. This discovery challenged the previously held view that affinity is the most important consideration for molecular glue design. Working with collaborators to investigate molecular glues as potential cancer therapeutics a similar study looked at drug stabilisation of inhibitory complexes of mouse double minute 2 (MDM2), a key negative regulator of the tumour suppressor protein p53 [24].

Understanding modes of action of antibiotics is also critical for understanding how resistance mechanisms play out for different bacterial proteins. Real-time reaction monitoring of bacterial membrane phosphatases, in the presence of antibiotics, highlights possible approaches to understanding their mode of action [25]. This approach found that two antibiotics (bacitracin and teixobactin) functioned by outcompeting the membrane-associated enzymes (UppP and PgpB) for substrates. These two membrane enzymes exhibit different lipid-binding behaviour, however, which could point to subtle but important differences in their cellular modes of action.

### Deciphering protein-small molecule interactions

Following successful target identification and validation, subsequent drug discovery steps often involve high-throughput screening (HTS) for hit discovery, hit-to-lead development, and lead optimisation. These stages traditionally rely upon either cellular assays or *in silico* docking. The major drawbacks of these methods are their indirect measurement of proteinedrug interactions, and reliance on static structures, respectively. Direct and in-depth characterisation of small molecule interactions, in an environment that captures the complexity and flexibility of their biological context, would therefore provide complementary information for the field. Capturing protein-ligand binding is possible by means of nMS by observing direct binding through addition of mass. In many cases this enables determination of the stoichiometry of exogenous and endogenous small molecule binding (>40 Da) within protein complexes in the hundreds of kDa range. Direct observation of protein–drug binding is therefore possible, even when using extensive small molecule libraries, providing the ligands have been organised such that they separated by mass. Challenges arise, however, when screening unknown libraries of diverse natural products, or in cases where library components are not as anticipated. These situations often arise when the active molecule is in fact a metabolite of an original library compound, the library compound does not match the expected structure from its synthesis, or additional allosteric modulation is found. Here we focus on the latest developments and recommend those looking for a more fundamental introduction to consult a recent article in *Chemical Reviews* [26].

HTS using both nanoflow needle and chip-based nMS methods have been applied to drug libraries, natural products, and fragments for several disease-linked target proteins. For example, using 96 well plate nMS technology, 133 carboxylic and tetrazole fragments were screened for their ability to disrupt HOP-HSP90 PPIs, linked to tumour malignancy [27]. A family of soluble candidate fragments were identified and proposed as a starting point for the development of new anti-cancer therapies. Rapid screening of 96 small molecules against SIRT5 was similarly performed using a microdroplet nMS system. In this case, 20 novel SIRT5 binders were identified which act as inhibitors by stabilising protein conformation [28]. Combining nMS with ion mobility (IM) can further enhance these screening approaches. By combining enhanced declustering with IM-enabled native top-down identification of novel hit compounds against the membrane-bound PfMATE protein was achieved from multiplexed ligand libraries [29]. Although these libraries are relatively modest in size, scale up to much larger libraries is possible with increasing automation, minimising sample preparation.

Localising where specifically drug candidates bind their target protein is a further area of interest in the field of drug discovery. Whilst traditionally performed using computational docking methods or hydrogen-deuterium exchange (HDX), top-down dissociation in combination with nMS is an additional method which could be exploited to achieve similar results. This phenomenon was demonstrated for protein complexes of the antimetastatic metallodrug RAPTA-C, using collision-induced dissociation (CID) to localise where drug interactions occurred on each protein [30]. The authors then went on to explore the use of IM and collision-induced unfolding (CIU) in combination with nMS to analyse alterations to protein folding induced by RAPTA-C binding. Similarly, nMS combined with electron capture dissociation (ECD) has been applied to localise calmodulin-ligand binding sites for individual protein conformations [31].

Developments in localising targeteligand interfaces are particularly exciting when considered in the context of allostery. The study of allostery has been reported in several instances, with a recent publication studying the allosteric effects of small molecule inhibitors on the CDK12/CDK13-Cyclin K complex [32]. It was found that one particular inhibitor, SR-4835 acted through a previously unknown method of allosteric activation, enabling it to destabilise the protein complex and modulate phosphorylation. The impact of allosteric catalytic core regulators on the 20 S proteasome has also been characterised using nMS [33]. The allosteric pathway discovered was found to propagate through the non-catalytic subunit PSMB4 rather than the enzymatic subunits, ultimately enabling the team to design a novel allosteric modulator.

Quantitative information on protein-ligand interactions, including enthalpic contributions to binding, can be determined by assessing the extent of drug binding as a function of temperature. This so-called variable-temperature nMS was used to characterise the response of cofactors binding to individual structures of myohemerythrin [34]. By contrast, the study of protein-ligand interactions on fast time scales (seconds to milliseconds) was enabled by temperature-jump nMS [35,36]. Kinetics, including drug residency times and dissociation constants (K_D_), can similarly be accessed by nMS for small-molecule protein complexes mediated by polar and electrostatic interactions [26]. Kinetic analysis of inhibitor affinities for the catabolic enzyme IDO1 was reported using both chip-based MS and size exclusion (SEC) approaches [37]. Limitations remain in MS applications to solely hydrophobically-mediated interactions, which can be more labile in the gas phase. However, given the low propensity for lipophilic molecules to become drug candidates this limitation can be viewed in a positive light since nMS HTS screens provide an implicit bias towards electrostatic interactions.

**Figure 3:**
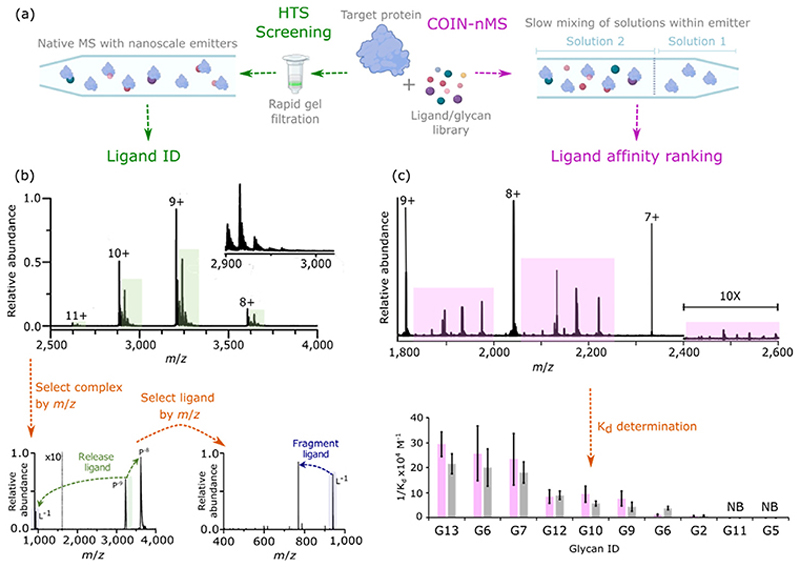
**(a)** Schematic showing the workflows used for high throughput natural product screening (left) and COIN-nMS (right); **(b)** native mass spectrum of human carbonic anhydrase I and crude natural product extracts from red onion peel containing 838−3336 compounds (top). Application of the multi-stage MS workflow to protein−ligand complexes formed between human carbonic anhydrase I and an unknown molecules from an ethanolic eucalyptus leaf extract (bottom). The ligand bound to carbonic anhydrase I was identified as a tannin ligand, 1,2,3,4,6-penta-O-galloyl-β-D-glucose; **(c)** COIN-nMS spectrum of GAL-3C with a heterogeneous glycan mixture containing known binders and non-binders at non-uniform concentrations (top). By monitoring signal intensity over time, K_d_ values were determined for individual glycans (bottom–purple bars), which are consistent with the values obtained using a traditional nMS approach (bottom–grey bars). Figure adapted with permission from Nguyen, G. T. H. H.; Bennett, J. L.; Liu, S.; Hancock, S. E.; Winter, D. L.; Glover, D. J.; Donald, W. A. Multiplexed screening of thousands of natural products for protein–ligand binding in native mass spectrometry. J. Am. Chem. Soc. 2021, 143 (50), 21,379–21387. Figure also adapted with permission from Bui, D. T.; Favell, J.; Kitova, E. N.; Li, Z.; McCord, K. A.; Schmidt, E. N.; Mozaneh, F.; Elaish, M.; El-Hawiet, A.; St-Pierre, Y.; Hobman, T. C.; Macauley, M. S.; Mahal, L. K.; Flynn, M. R.; Klassen, J. S. Absolute affinities from quantitative shotgun glycomics using concentration-independent (COIN) native mass spectrometry. ACS Cent. Sci. 2023, 9 (7), 1374–1387.

Some of the most exciting recent breakthroughs in applying nMS to protein-small molecule interactions employ the HTS of hundreds to thousands of molecules in combination with in-depth quantitative and qualitative insights. This phenomenon was demonstrated by screening natural products from five biological sources (red onion peel, red clover, parsley, eucalyptus leaves and orange peel) against human carbonic anhydrase 1, a target for glaucoma, epilepsy, obesity, and tumours, Figure 3(a). In an impressive demonstration, as many as 8900 ligands were examined in a single experiment lasting just a few minutes [38]. By harnessing 250 nm emitters rather than the standard 2000 nm size, similar molecular weight complexes were resolved, enabling identification and stoichiometry of bound ligands to be defined, Figure 3(b). To achieve this, multistage MS was performed on the protein-ligand complexes, exploiting fragmentation approaches analogous to those previously reported in native-omics and collision-induced affinity section (CIAS) workflows [39,40]. Screening non-uniform glycan mixtures against different lectin proteins, using a concentration-independent (COIN)-nMS method, Figure 3(a) [41] allowed direct observation of binding between the two entities. By harnessing slow mixing within a nanoscale emitter, it was possible to achieve an ever-changing concentration gradient at the emitter tip. Simultaneous K_D_ determination for multiple protein-glycan complexes was therefore possible despite unknown concentrations of the original glycan solutions, Figure 3(c).

Understanding off-target drug binding and its subsequent biological effects is an additional area where nMS has the potential to contribute by providing insights into toxicity. Theoretically this could be achieved on a bulk scale using the native top-down proteomics method previously applied to study mouse hearts and human cancer cell lines [42]. Incorporation of drug treatment into this workflow remains unexplored but could prove transformative in terms of identifying widescale promiscuous drug binding. A recent targeted nMS approach has proven successful in revealing off-target drug binding of two phosphodiesterase inhibitors, vardenafil and sildenafil, whose intended target is phosphodiesterase 5 (PDE5) [43]. By studying the action of these drugs in a native cell signalling environment, their preferential off-target binding to membrane-associated proteoforms of G-proteins was observed, implying that hydrophobic modifications enhance that off-target binding. In particular, it was noted that the farnesyl and geranyl–geranyl modifications on PDE6α and PDE6β are proximal to the sites at which vardenafil binds, potentially creating a conduit for the drug to interact hydrophobically with the protein and the modified G-proteins before binding to the catalytic site.

### Integration with traditional structural biology

High-resolution structural techniques, notably cryogenic electron microscopy (cryo-EM) and X-ray crystallography (XRC), currently enable both target validation and *in silico* hit identification as part of the drug discovery pipeline [44]. Despite their power, these methods remain time-intensive and expensive endeavours, making obtaining high-quality protein samples prior to analysis key. The ability of nMS to assist structural biology was demonstrated as early as 2014, where optimised lipid stabilisation enabled a 2.3 Å X-ray structure of AmtB to be obtained [45]. Over the last five years, there has been an even greater push towards developing nMS as a screening method for sample integrity and purity ahead of imaging. Using minimal protein, one research team devised such a system, with an interrogation time per sample of only 20 min [46]. As innovations in this area continue, likely making use of the high throughput approaches discussed earlier in this review, they offer exciting promise for improving structure-led drug design.

A novel alternative approach to the sample quality problem in structural biology is evidenced in recent exciting developments using nMS as a protein preparation platform for cryo-EM. Whilst this integrative concept has been around for some time, recent advancements in both fields have made progress over the last three years, particularly fruitful [47–50]. Current approaches, use quadrupole Orbitrap instruments with modifications, including soft landing stages added *in house* [51,52]. Early efforts used these platforms to land native proteins on EM grids at room temperature prior to imaging with negative stain and cryo-EM [53–56]. In doing so, the teams were able to obtain structures, including protein complexes GroEL, and ferritin in both holo and apo states. The instrument setup is the key in this achievement, with quadrupole selection allowing clean isolation of individual molecular entities, therefore providing improved sampling compared to traditional workflows.

Following on from these pioneering efforts, attention turned towards adapting the instrumentation for cryodeposition [54,57,58]. Under optimised conditions, ice layers of desired phase and thickness were grown *in situ*, avoiding the potential pitfalls associated with traditional plunge freezing. Averaging of 400,000 single particle images collected in this way resulted in a 2.6 Å resolution structure of β-galactosidase [58]. Only minor dehydration-driven compaction of β-galactosidase was observed, compared to plunge freezing, confirming the relevance of gas phase nMS to solution state biology.

These novel developments, integrating nMS with cryo-EM, suggest exciting new opportunities for the field of drug discovery. Enabling their high-resolution image of challenging drug targets could prove highly informative for *in silico* docking, while the mass selection capabilities offer tantalising opportunities for selecting complexes with bound drugs.

## Concluding remarks

Technologies for studying target-small molecule interactions by nMS have already been successfully leveraged by pharmaceutical companies. By measuring proteinedrug interactions directly using minimal protein material and at high throughput means that this approach has the potential to be transformative in identifying hits and optimising them into lead compounds. As the method is more widely adopted, we predict that insights into drug modes of action and off-target binding will also be incorporated into the work-flow, furthering its informative power.

A key requirement for wider uptake, however, is increased nMS accessibility through communication between disciplines, training and commercialisation of user-friendly instrumentation and analysis tools. Additional developments to address current nMS limitations, which include the retention of labile small-molecule binding, particularly in the context of membrane proteins, where liberating the protein from the membrane mimetic often simultaneously releases the ligand. We have no doubt, however, that just as the field continues to be revolutionised by fundamental developments in instrumentation and assignment strategies, so too will further approaches emerge with respect to ligand binding. These may well include DNA encoded libraries coupled with affinity-based MS approaches [59].

Looking to the future, as we continue to evolve ways to better emulate cell-based assays, while maintaining the intricate complexity of endogenous protein systems, the unique selectivity and fragmentation capabilities of MS will become increasingly important. This dual potential of nMS, in reflecting the biological relevance of a cell-based assay while exploiting mass resolution to capture direct binding interactions, is a particularly compelling prospect. Moreover, enhancing nMS to include improved fragmentation capabilities not only defines small molecule interactors but also enables characterisation of proteoform diversity, accounting for off-target binding and proteoform selectivity. This expanded application landscape holds immense promise for advancing our understanding of complex biological systems. By providing molecular-level insights into interactions within a cell-based context, we hope that nMS will pave the way for transformative discoveries in the field of drug discovery.

## Data Availability

No data was used for the research described in the article.

## References

[1] Drews J (1979). Drug discovery: a historical perspective. Science.

[2] Tamara S, den Boer MA, Heck AJR (2022). High-resolution native mass spectrometry. Chem Rev.

[3] Keener JE, Zhang G, Marty MT (2021). Native mass spectrometry of membrane proteins. Anal Chem.

[4] Bunnage ME (2011). Getting pharmaceutical R&D back on target. Nat Chem Biol.

[5] Rogawski R, Sharon M (2022). Characterizing endogenous protein complexes with biological mass spectrometry. Chem Rev.

[6] Hale OJ, Hughes JW, Sisley EK, Cooper HJ (2022). Native ambient mass spectrometry enables analysis of intact endogenous protein assemblies up to 145 kDa directly from tissue. Anal Chem.

[7] Sisley EK, Hale OJ, Styles IB, Cooper HJ (2022). Native ambient mass spectrometry imaging of ligand-bound and metal-bound proteins in rat brain. J Am Chem Soc.

[8] Yang M, Hu H, Su P, Thomas PM, Camarillo JM, Greer JB, Early BP, Fellers RT, Kelleher NL, Laskin J (2022). Proteoform-Selective imaging of tissues using mass spectrometry. Angew Chem Int Ed.

[9] Rogawski R, Rogel A, Bloch I, Gal M, Horovitz A, London N, Sharon M (2021). Intracellular protein–drug interactions probed by direct mass spectrometry of cell lysates. Angew Chem Int Ed.

[10] Illes-Toth E, Hale OJ, Hughes JW, Strittmatter N, Rose J, Clayton B, Sargeant R, Jones S, Dannhorn A, Goodwin RJA (2022). Mass spectrometry detection and imaging of a non-covalent protein–drug complex in tissue from orally dosed rats. Angew Chem.

[11] Chorev DS, Tang H, Rouse SL, Bolla JR, von Kügelgen A, Baker LA, Wu D, Gault J, Grünewald K, Bharat TAM (2020). The use of sonicated lipid vesicles for mass spectrometry of membrane protein complexes. Nat Protoc.

[12] Panda A, Giska F, Duncan AL, Welch AJ, Brown C, McAllister R, Hariharan P, Goder JND, Coleman J, Ramakrishnan S (2023). Direct determination of oligomeric organization of integral membrane proteins and lipids from intact customizable bilayer. Nat Methods.

[13] Zhu Y, Yun SD, Zhang T, Chang JY, Stover L, Laganowsky A (2023). Native mass spectrometry of proteoliposomes containing integral and peripheral membrane proteins. Chem Sci.

[14] Chen S, Getter T, Salom D, Wu D, Quetschlich D, Chorev DS, Palczewski K, Robinson CV (2022). Capturing a rhodopsin receptor signalling cascade across a native membrane. Nature.

[15] Lutomski CA, El-Baba TJ, Hinkle JD, Liko I, Bennett JL, Kalmankar NV, Dolan A, Kirschbaum C, Greis K, Urner L (2023). Infrared multiphoton dissociation enables top-down characterization of membrane protein complexes and G protein-coupled receptors. Angew Chem Int Ed.

[16] Juliano BR, Keating JW, Ruotolo BT (2023). Infrared photoactivation enables improved native top-down mass spectrometry of transmembrane proteins. Anal Chem.

[17] Beveridge R, Kessler D, Rumpel K, Ettmayer P, Meinhart A, Clausen T (2020). Native mass spectrometry can effectively predict PROTAC efficacy. ACS Cent Sci.

[18] Sternicki LM, Nonomiya J, Liu M, Mulvihill MM, Quinn RJ (2021). Native mass spectrometry for the study of PROTAC GNE-987-containing ternary complexes. ChemMedChem.

[19] Song JH, Wagner ND, Yan J, Li J, Huang RYC, Balog AJ, Newitt JA, Chen G, Gross ML (2021). Native mass spectrometry and gas-phase fragmentation provide rapid and in-depth topological characterization of a PROTAC ternary complex. Cell Chem Biol.

[20] Ahmed IMM, Beveridge R (2023). Native mass spectrometry interrogation of complexes formed during targeted protein degradation. Rapid Commun Mass Spectrom.

[21] Bellamy-Carter J, Mohata M, Falcicchio M, Basran J, Higuchi Y, Doveston RG, Leney AC (2021). Discovering protein–protein interaction stabilisers by native mass spectrometry. Chem Sci.

[22] Verhoef CJA, Kay DF, van Dijck L, Doveston RG, Brunsveld L, Leney AC, Cossar PJ (2023). Tracking the mechanism of covalent molecular glue stabilization using native mass spectrometry. Chem Sci.

[23] Huang X, Kamadurai H, Siuti P, Ahmed E, Bennett JL, Donald WA (2023). Oligomeric remodeling by molecular glues revealed using native mass spectrometry and mass photometry. J Am Chem Soc.

[24] Ward JA, Romartinez-Alonso B, Kay DF, Bellamy-Carter J, Thurairajah B, Basran J, Kwon H, Leney AC, Macip S, Roversi P (2024). Characterizing the protein–protein interaction between MDM2 and 14-3-3s; proof of concept for small molecule stabilization. J Biol Chem.

[25] Oluwole AO, Hernández-Rocamora VM, Cao Y, Li X, Vollmer W, Robinson CV, Bolla JR (2024). Real-time biosynthetic reaction monitoring informs the mechanism of action of antibiotics. J Am Chem Soc.

[26] Bennett JL, Nguyen GTH, Donald WA (2022). Protein–small molecule interactions in native mass spectrometry. Chem Rev.

[27] Vaaltyn MC, Mateos-Jimenez M, Müller R, Mackay CL, Edkins AL, Clarke DJ, Veale CGL (2022). Native mass spectrometry-guided screening identifies hit fragments for HOP-HSP90 PPI inhibition. Chembiochem.

[28] D’Amico CI, Polasky DA, Steyer DJ, Ruotolo BT, Kennedy RT (2022). Ion mobility-mass spectrometry coupled to droplet microfluidics for rapid protein structure analysis and drug discovery. Anal Chem.

[29] Sokratous K, Cooper-Shepherd DA, Ujma J, Qu F, Giles K, Ben-Younis A, Hensen M, Langridge JI, Gault J, Jazayeri A (2024). Enhanced declustering enables native top-down analysis of membrane protein complexes using ion-mobility time-aligned fragmentation. J Am Soc Mass Spectrom.

[30] Eade L, Sullivan MP, Allison TM, Goldstone DC, Hartinger C (2024). Not all binding sites are equal: site determination and folding state analysis of gas-phase protein–metallodrug adducts. Chem Eur J.

[31] Britt HM, Ben-Younis A, Page N, Thalassinos K (2024). A conformation-specific approach to native top-down mass spectrometry. J Am Soc Mass Spectrom.

[32] Bai Y, Liu Z, Li Y, Zhao H, Lai C, Zhao S, Chen K, Luo C, Yang X, Wang F (2023). Structural mass spectrometry probes the inhibitor-induced allosteric activation of CDK12/CDK13-cyclin K dissociation. J Am Chem Soc.

[33] Deshmukh FK, Ben-Nissan G, Olshina MA, Füzesi-Levi MG, Polkinghorn C, Arkind G, Leushkin Y, Fainer I, Fleishman SJ, Tawfik D (2023). Allosteric regulation of the 20S proteasome by the catalytic core regulators (CCRs) family. Nat Commun.

[34] Woodall DW, El-Baba TJ, Fuller DR, Liu W, Brown CJ, Laganowsky A, Russell DH, Clemmer DE (2019). Variable-temperature ESI-IMS-MS analysis of myohemerythrin reveals ligand losses, unfolding, and a non-native disulfide bond. Anal Chem.

[35] Woodall DW, El-Baba TJ, Fuller DR, Liu W, Brown CJ, Laganowsky A, Russell DH, Clemmer DE (2019). Variable-temperature ESI-IMS-MS analysis of myohemerythrin reveals ligand losses, unfolding, and a non-native disulfide bond. Anal Chem.

[36] Marchand A, Czar MF, Eggel EN, Kaeslin J, Zenobi R (2020). Studying biomolecular folding and binding using temperature-jump mass spectrometry. Nat Commun.

[37] Ren C, Bailey AO, Vanderporten E, Oh A, Phung W, Mulvihill MM, Harris SF, Liu Y, Han G, Sandoval W (2019). Quantitative determination of protein-ligand affinity by size exclusion chromatography directly coupled to high-resolution native mass spectrometry. Anal Chem.

[38] Nguyen GTHH, Bennett JL, Liu S, Hancock SE, Winter DL, Glover DJ, Donald WA (2021). Multiplexed screening of thousands of natural products for protein–ligand binding in native mass spectrometry. J Am Chem Soc.

[39] Gault J, Liko I, Landreh M, Shutin D, Bolla JR, Jefferies D, Agasid M, Yen HY, Ladds MJGW, Lane DP (2020). Combining native and ‘omics’ mass spectrometry to identify endogenous ligands bound to membrane proteins. Nat Methods.

[40] Gu Y, Liu M, Ma L, Quinn RJ (2024). Identification of ligands for ion channels: trpm2. Chembiochem.

[41] Bui DT, Favell J, Kitova EN, Li Z, McCord KA, Schmidt EN, Mozaneh F, Elaish M, El-Hawiet A, St-Pierre Y (2023). Absolute affinities from quantitative Shotgun Glycomics using concentration-independent (COIN) native mass spectrometry. ACS Cent Sci.

[42] Skinner OS, Haverland NA, Fornelli L, Melani RD, Do Vale LHF, Seckler HS, Doubleday PF, Schachner LF, Srzenti’c K, Kelleher NL (2017). Top-down characterization of endogenous protein complexes with native proteomics. Nat Chem Biol.

[43] Lutomski CA, Bennett JL, El-Baba TJ, Wu D, Hinkle JD, Burnap SA, Like I, Mullen C, Syka JEP, Struwe WB (2024). Defining proteoform-specific interactions for drug targeting in a native cell signalling environment. Nat Chem.

[44] Nasim F, Qureshi IA (2022). Role of structural biology methods in drug discovery. Advances in protein molecular and structural biology methods.

[45] Laganowsky A, Reading E, Allison TM, Ulmschneider MB, Degiacomi MT, Baldwin AJ, Robinson CV (2014). Membrane proteins bind lipids selectively to modulate their structure and function. Nature.

[46] Olinares PDB, Kang JY, Llewellyn E, Chiu C, Chen J, Malone B, Saecker RM, Campbell EA, Darst SA, Chait BT (2021). Native mass spectrometry-based screening for optimal sample preparation in single-particle cryo-EM. Structure.

[47] Westphall MS, Lee KW, Salome AZ, Coon JJ, Grant T (2023). Mass spectrometers as cryoEM grid preparation instruments. Curr Opin Struct Biol.

[48] Franchetti V, Solka BH, Baitinger WE, Amy JW, Cooks RG (1977). Soft landing of ions as a means of surface modification. Int J Mass Spectrom Ion Phys.

[49] Mikhailov VA, Mize TH, Benesch JLP, Robinson CV (2014). Mass-selective soft-landing of protein assemblies with controlled landing energies. Anal Chem.

[50] Benesch JLP, Ruotolo BT, Simmons DA, Barrera NP, Morgner N, Wang L, Saibil HR, Robinson CV (2010). Separating and visualising protein assemblies by means of preparative mass spectrometry and microscopy. J Struct Biol.

[51] Fremdling P, Esser TK, Saha B, Makarov AA, Fort KL, Reinhardt-Szyba M, Gault J, Rauschenbach S (2022). A preparative mass spectrometer to deposit intact large native protein complexes. ACS Nano.

[52] Westphall MS, Lee KW, Salome AZ, Lodge JM, Grant T, Coon JJ (2022). Three-dimensional structure determination of protein complexes using matrix-landing mass spectrometry. Nat Commun.

[53] Esser TK, Böhning J, Fremdling P, Agasid MT, Costin A, Fort K, Konijnenberg A, Gilbert JD, Bahm A, Makarov A (2022). Mass-selective and ice-free electron cryomicroscopy protein sample preparation via native electrospray ion-beam deposition. PNAS Nexus.

[54] Esser TK, Böhning J, Fremdling P, Bharat T, Gault J, Rauschenbach S (2022). Cryo-EM samples of gas-phase purified protein assemblies using native electrospray ion-beam deposition. Faraday Discuss.

[55] Salome AZ, Lee KW, Grant T, Westphall MS, Coon JJ (2022). Matrix-landing mass spectrometry for electron microscopy imaging of native protein complexes. Anal Chem.

[56] Lee KW, Salome AZ, Westphall MS, Grant T, Coon JJ (2023). Onto grid purification and 3D reconstruction of protein complexes using matrix-landing native mass spectrometry. J Proteome Res.

[57] Westphall MS, Lee KW, Hemme C, Salome AZ, Mertz K, Grant T, Coon JJ (2023). Cryogenic soft landing improves structural preservation of protein complexes. Anal Chem.

[58] Esser TK, Böhning J, Önür A, Chinthapalli DK, Eriksson L, Grabarics M, Fremdling P, Konijnenberg A, Makarov A, Botman A (2024). Cryo-EM of soft-landed β-galactosidase: gas-phase and native structures are remarkably similar. Sci Adv.

[59] Xia B, Franklin GJ, Lu X, Bedard KL, Grady LC, Summerfield JD, Shi EX, King BW, Lind KE, Chiu C (2021). DNA-encoded library hit confirmation: bridging the gap between on-DNA and off-DNA chemistry. ACS Med Chem Lett.

